# *Burkholderia thailandensis *harbors two identical *rhl *gene clusters responsible for the biosynthesis of rhamnolipids

**DOI:** 10.1186/1471-2180-9-263

**Published:** 2009-12-17

**Authors:** Danielle Dubeau, Eric Déziel, Donald E Woods, François Lépine

**Affiliations:** 1 INRS-Institut Armand Frappier, Laval, Québec, H7V 1B7, Canada; 2 Department of Microbiology and Infectious Diseases, Faculty of Medicine, University of Calgary Health Sciences Centre, Calgary, Alberta, T2N 4N1, Canada

## Abstract

**Background:**

Rhamnolipids are surface active molecules composed of rhamnose and β-hydroxydecanoic acid. These biosurfactants are produced mainly by *Pseudomonas aeruginosa *and have been thoroughly investigated since their early discovery. Recently, they have attracted renewed attention because of their involvement in various multicellular behaviors. Despite this high interest, only very few studies have focused on the production of rhamnolipids by *Burkholderia *species.

**Results:**

Orthologs of *rhlA*, *rhlB *and *rhlC*, which are responsible for the biosynthesis of rhamnolipids in *P. aeruginosa*, have been found in the non-infectious *Burkholderia thailandensis*, as well as in the genetically similar important pathogen *B. pseudomallei*. In contrast to *P. aeruginosa*, both *Burkholderia *species contain these three genes necessary for rhamnolipid production within a single gene cluster. Furthermore, two identical, paralogous copies of this gene cluster are found on the second chromosome of these bacteria. Both *Burkholderia *spp. produce rhamnolipids containing 3-hydroxy fatty acid moieties with longer side chains than those described for *P. aeruginosa*. Additionally, the rhamnolipids produced by *B. thailandensis *contain a much larger proportion of dirhamnolipids versus monorhamnolipids when compared to *P. aeruginosa*. The rhamnolipids produced by *B. thailandensis *reduce the surface tension of water to 42 mN/m while displaying a critical micelle concentration value of 225 mg/L. Separate mutations in both *rhlA *alleles, which are responsible for the synthesis of the rhamnolipid precursor 3-(3-hydroxyalkanoyloxy)alkanoic acid, prove that both copies of the *rhl *gene cluster are functional, but one contributes more to the total production than the other. Finally, a double Δ*rhlA *mutant that is completely devoid of rhamnolipid production is incapable of swarming motility, showing that both gene clusters contribute to this phenotype.

**Conclusions:**

Collectively, these results add another *Burkholderia *species to the list of bacteria able to produce rhamnolipids and this, by the means of two identical functional gene clusters. Our results also demonstrate the very impressive tensio-active properties these long-chain rhamnolipids possess in comparison to the well-studied short-chain ones from *P. aeruginosa*.

## Background

Rhamnolipids are surface-active compounds that have been extensively studied since their early identification in *Pseudomonas aeruginosa *cultures in the late 1940s [1]. However, it was only in the mid 1960s that the structure of a rhamnolipid molecule was first reported [2]. Due to their excellent tensioactive properties, low toxicity and high biodegradability, these biosurfactants are promising candidates for a variety of industrial applications as well as bioremediation processes [3,4]. Furthermore, rhamnolipids have recently received renewed attention because of their involvement in *P. aeruginosa *multicellular behavior, such as biofilm development and swarming motility [5-7]. Rhamnolipids are also considered virulence factors as they interfere with the normal functioning of the tracheal ciliary system and are found in sputa of cystic fibrosis (CF) patients infected by *P. aeruginosa *[8-10]. Moreover, rhamnolipids inhibit the phagocytic response of macrophages and are known as the heat-stable extracellular hemolysin produced by *P. aeruginosa *[11,12].

These amphiphilic molecules are usually produced by *P. aeruginosa *as a complex mixture of congeners composed of one or two molecules of L-rhamnose coupled to a 3-hydroxyalkanoic acid dimer, the most abundant being L-rhamnosyl-3-hydroxydecanoyl-3-hydroxydecanoate (Rha-C_10_-C_10_) and L-rhamnosyl-L-rhamnosyl-3-hydroxydecanoyl-3-hydroxydecanoate (Rha-Rha-C_10_-C_10_) [13-15]. The biosynthetic pathway of rhamnolipids has been the subject of many studies that have demonstrated the implication of three crucially important genes, *rhlA, rhlB *and *rhlC*. The first enzyme, RhlA, is responsible for the interception of two molecules of β-hydroxydecanoyl-ACP, an intermediate in the *de novo *fatty acid biosynthesis cycle, to produce 3-hydroxyalkanoic acid dimers, known as 3-(3-hydroxyalkanoyloxy)alkanoic acids (HAAs) [16,17]. The second reaction, implicating the membrane-bound RhlB rhamnosyltransferase, uses dTDP-L-rhamnose to add the first rhamnose moiety to an HAA molecule, thus forming a monorhamnolipid (L-rhamnosyl-3-hydroxyalkanoyl-3-hydroxyalkanoate). Finally, an additional rhamnosyltransferase, RhlC, couples a second rhamnose molecule to a monorhamnolipid by the means of another dTDP-L-rhamnose, producing the final dirhamnolipid (L-rhamnosyl-L-rhamnosyl-3-hydroxyalkanoyl-3-hydroxyalkanoate) [18,19].

Previously assigned to the *Pseudomonas *genus, *Burkholderia *spp. are attracting increasing interest because of their involvement in human infections. *Burkholderia *is best known for its pathogenic members like *B. pseudomallei*, the causative agent of melioidosis, as well as the opportunistic pathogens belonging to the *B. cepacia *complex [20,21]. Two studies have reported evidence of the production of a single dirhamnolipid by *B. pseudomallei *as well as by another member of the same genus, *B. plantarii *[22,23]. Here, we investigate the production of rhamnolipids by *B. thailandensis*, a non-infectious *Burkholderia *species closely related to *B. pseudomallei *[24], and by *B. pseudomallei *itself. In contrast to the mandated *B. pseudomallei *guidelines, an advantage to studying *B. thailandensis *is that it does not require biosafety level 3 conditions, and there is no restriction on the use of antibiotic-resistance markers for its genetic manipulation. In addition, numerous studies have shown to what extreme level these two *Burkholderia *species are closely related from a genetic point of view and that *B. thailandensis *can serve as a surrogate for studying many different traits, including physiological characteristics as well as pathogenic factors in regards to *B. pseudomallei *[25,26].

## Results

### Presence of *rhlABC *homologs in *B. thailandensis *and *B. pseudomallei*

**Figure 1:**
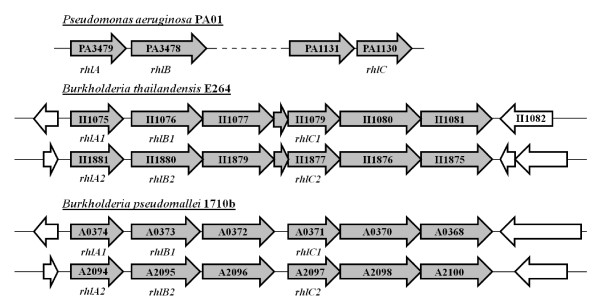
**Genetic arrangement of *rhlA*, *rhlB *and *rhlC *in the genomes**. Schematic representation of the bicistronic *P. aeruginosa *PAO1 http://www.pseudomonas.com regions containing the *rhlAB *and *rhlC *genes as well as the two identical gene clusters containing the homologous *rhlA, rhlB *and *rhlC *genes in *B. thailandensis *E264 and *B. pseudomallei *1710b http://www.burkholderia.com.

Following a nucleotide and protein similarity search using algorithms blastn and blastp with standard parameters http://blast.ncbi.nlm.nih.gov/Blast.cgi, respectively, in sequenced *B. thailandensis *and *B. pseudomallei *genome sequences, close orthologs of the *P. aeruginosa *rhamnolipid-biosynthesis genes *rhlA, rhlB *and *rhlC *were found in all associated strains as gene clusters. Interestingly, both species possess two 100% identical *rhl *gene clusters on their second chromosome (Figure 1). A search in the partially sequenced genome of *B. pseudomallei *1026b (Genomes OnLine Database; http://www.genomesonline.org), the strain used in this study, indicated the same arrangement as in the completely sequenced strain 1710b. The *rhlA*/*rhlB*/*rhlC *orthologs of these two *Burkholderia *species are highly similar to one another with nucleotide identity ranging from 89% to 96%. Furthermore, the protein encoded by these genes share almost 50% identity with those of *P. aeruginosa *PAO1, which possesses a single copy of these genes on its genome. Another interesting observation is that for *P. aeruginosa*, *rhlA *and *rhlB *are found in one operon whereas *rhlC *is found in a different bicistronic operon (Figure 1). Finally, Table 1 shows that the remaining ORFs present in the *rhl *gene clusters, including the one adjacent to *rhlC *in *P. aeruginosa*, all seem to have functions related to transport or efflux.

**Table 1:** Predicted functions of the remaining ORFs

Gene annotation	Predicted function^1^
PA1131	Probable Major Facilitator Superfamily (MFS) Transporter

BTH_II1077/BTH_II1879	Drug Resistance Transporter, EmrB/QacA Family
BTH_II1078/BTH_II1878	Hypothetical Protein
BTH_II1080/BTH_II1876	RND Efflux System, Outer Membrane Lipoprotein, NodT Family

BTH_II1081/BTH_II1875	Multidrug Resistance Protein (EmrA)
BURPS1710b_0372/BURPS1710b_2096	Multidrug Resistance Protein (BcrA)
BURPS1710b_0370/BURPS1710b_2098	RND Efflux System, Outer Membrane Lipoprotein, NodT Family
BURPS1710b_0368/BURPS1710b_2100	Multidrug Resistance Protein (EmrA)

^1 ^Predicted functions from http://www.pseudomonas.com and http://www.burkholderia.com.

Predicted functions of the remaining ORFs present in the *rhl *gene cluster in *B. thailandensis *and *B. pseudomallei*, including the one adjacent to *rhlC *in *P. aeruginosa*.

### Rhamnolipid production by *B. thailandensis *and *B. pseudomallei*

Due to the high similarity between the *rhlA*/*rhlB*/*rhlC *genes found in *P. aeruginosa *and their homologs in *B. thailandensis*, the latter was tested for the production of rhamnolipids. Using *B. thailandensis *in various rhamnolipid production growth conditions, the initial results from liquid chromatography/mass spectrometry (LC/MS) analysis revealed a dominant peak in the total-ion chromatograph (TIC). This peak presented a pseudomolecular ion of *m/z *761 in negative-ion mode, a value that is compatible with a compound consisting of two L-rhamnose molecules as well as two β-hydroxytetradecanoic acids. A corresponding rhamnolipid, 2-*O*-α-L-rhamnopyranosyl-α-L-rhamnopyranosyl-β-hydroxytetradecanoyl-β-hydroxytetradecanoate (Rha-Rha-C_14_-C_14_), with a molecular weight of 762 Da, has been previously reported from *B. pseudomallei *and *B. plantarii *cultures [22,23,27]. Further experimentation was done to investigate the effects of various growth media and carbon sources on the production of rhamnolipids by *B. thailandensis*. Mineral and rich culture media were assayed: tested substrates included carbohydrates such as mannitol, dextrose, sucrose, glycerol and fructose along with various vegetable oils such as canola oil, olive oil, palm oil and sunflower oil, all at a final concentration of 4% (data not shown). Several studies using plant-derived oils have demonstrated that these inexpensive hydrophobic materials are excellent carbon substrates for biosurfactant production by *P. aeruginosa *[28,29]. Under our experimental conditions, glycerol and canola oil were the best carbohydrate and vegetable oil for rhamnolipid production, achieving concentrations of 419.10 mg/L and 1473.72 mg/L, respectively, after 13 days of culture (Table 2). In both cases, the dirhamnolipid Rha-Rha-C_14_-C_14 _was the most abundant with values ranging from 70% to 77% relative to total rhamnolipids, while its precursor Rha-C_14_-C_14 _dominates the monorhamnolipid category with 5.8 and 6.5% of total rhamnolipids. Detailed analysis of *B. thailandensis *cultures revealed a series of long chain rhamnolipids, as shown in Table 2. These rhamnolipids are predominately composed of a C_14_-C_14 _chain length fatty acid moiety as well as others comprised of chains ranging from C_10_-C_12 _to C_16_-C_16 _chain length.

**Table 2:** Maximal production and relative abundance of the HAAs and rhamnolipids produced by *B. thailandensis *E264

HAA/Rhamnolipid	Pseudomolecular ion	Production (mg/L)		Relative abundance (%)^1^	
		
		Glycerol	Canola oil	Glycerol	Canola oil
C_10_-C_12_	385	N/D^2^	4.59	-	-
C_12_-C_12_	413	N/D	N/D	-	-
C_12_-C_14_	441	N/D	N/D	-	-
C_14_-C_14_	469	N/D	N/D	-	-
C_14_-C_16_	497	N/D	N/D	-	-
C_16_-C_16_	525	1.60	7.05	-	-

Rha-C_10_-C_12_	531	N/D	0.98	0.00	0.07
Rha-C_12_-C_12_	559	0.57	6.48	0.14	0.44
Rha-C_12_-C_14_	587	1.86	13.75	0.45	0.94
Rha-C_14_-C_14_	615	24.37	94.53	5.84	6.47
Rha-C_14_-C_16_	643	1.16	5.42	0.28	0.37
Rha-C_16_-C_16_	671	N/D	N/D	0.00	0.00

Rha-Rha-C_10_-C_12_	677	0.75	7.44	0.18	0.51
Rha-Rha-C_12_-C_12_	705	7.41	49.43	1.77	3.38
Rha-Rha-C_12_-C_14_	733	28.48	179.73	6.82	12.29
Rha-Rha-C_14_-C_14_	761	321.42	1021.20	76.99	69.85
Rha-Rha-C_14_-C_16_	789	31.24	82.37	7.48	5.63
Rha-Rha-C_16_-C_16_	817	0.26	0.73	0.06	0.05

Total		419.10	1473.72		

^1 ^Relative abundance of rhamnolipids only.

^2 ^N/D: Not detected.

Cultures were grown on 4% glycerol and canola oil as respective carbon sources. LC/MS analysis was performed after 13 days of incubation at 37°C.

**Figure 2:**
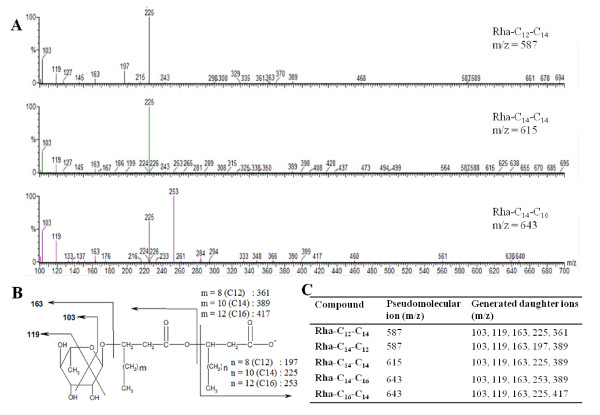
**Congener analysis of rhamnolipids from *B. thailandensis***. A) Mass spectra of the fragmented *m/z *587, 615 and 643 pseudomolecular ions of congeners Rha-C_12_-C_14_, Rha-C_14_-C_12_, Rha-C_14_-C_14_, Rha-C_14_-C_16 _and Rha-C_16_-C_14_. B) Schematic representation of observed fragmentation patterns of a monorhamnolipid. C) Daughter ions generated by fragmentation of the specified congeners.

To confirm that the ions identified by LC/MS are indeed rhamnolipids, they were fragmented and analyzed by tandem mass spectrometry (LC/MS/MS). To allow for comparison with *P. aeruginosa *rhamnolipids, monorhamnolipids obtained from *B. thailandensis *were fragmented and the observed fragmentation pattern was similar to the one we observed for *P. aeruginosa *[13]. For an isomeric pair of rhamnolipid congeners bearing two 3-hydroxy fatty acids of different chain lengths (for example Rha-C_12_-C_14 _and Rha-C_14_-C_12_), the relative abundance of the various congeners was studied. Because such isomers cannot be resolved chromatographically, the LC/MS/MS spectrum of their common pseudomolecular ions show fragment ions characteristic of both compounds, as seen in Figure 2. As we have previously reported for *P. aeruginosa *[14], within each isomeric pair, the rhamnolipid congener with the shortest chain adjacent to the sugar moiety is more abundant. To verify whether the rhamnolipids produced by *B. thailandensis *share this characteristic, they were subjected to an enzymatic hydrolysis of their rhamnose groups with naringinase [30] to produce the corresponding HAAs. The same stoichiometrical preference was confirmed.

**Figure 3:**
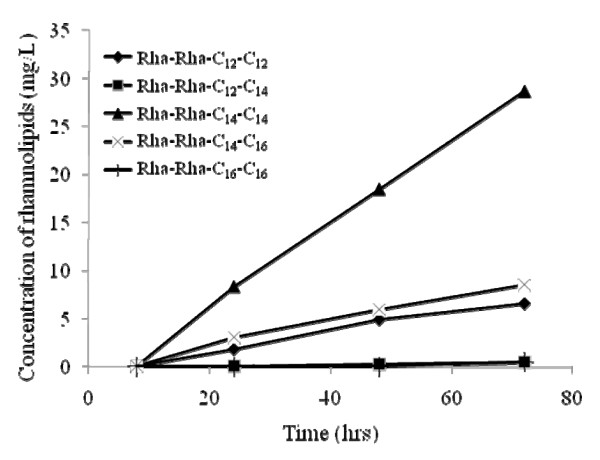
**Production of the most abundant dirhamnolipids in a *B. pseudomallei *1026b culture**. Bacteria were grown in NB supplemented with 4% glycerol as carbon source. Rhamnolipids were quantified by LC/MS.

With these results in hand, we investigated the potential of the highly genetically related species *B. pseudomallei *to produce a range of rhamnolipids other than the previously described Rha-Rha-C_14_-C_14_. Figure 3 shows the production of the most abundant rhamnolipids by this pathogen. The same long-chain bearing congeners found in *B. thailandensis *were also discovered in *B. pseudomallei*, including the dominant Rha-Rha-C_14_-C_14_.

### Critical Micelle Concentration (CMC) and surface tension assays

**Figure 4:**
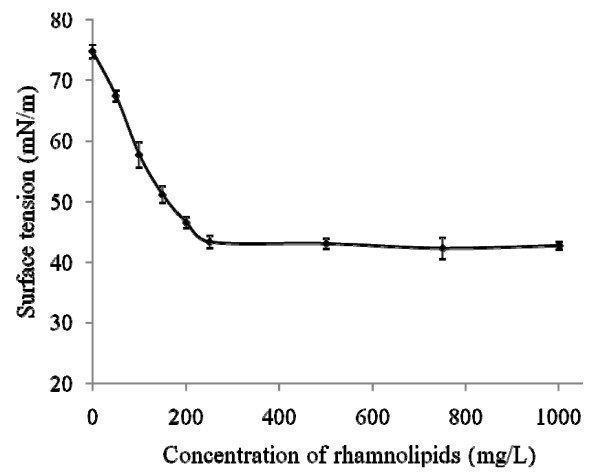
**Surface tension and CMC value**. Surface tension of the total mixture of rhamnolipids and HAAs extracted from a *B. thailandensis *E264 culture. Each data point shows the mean of triplicate measurements. Error bars represent the Standard Deviation (SD).

To investigate the potential of the long-chain rhamnolipids produced by *Burkholderia *species for lowering surface tension, the critical micelle concentration of this mixture of rhamnolipid congeners was established (Figure 4). At the CMC of about 250 mg/L, the surface tension is lowered to 43 mN/m.

### Both *rhlA *alleles are functional and necessary for maximal rhamnolipid production

**Figure 5:**
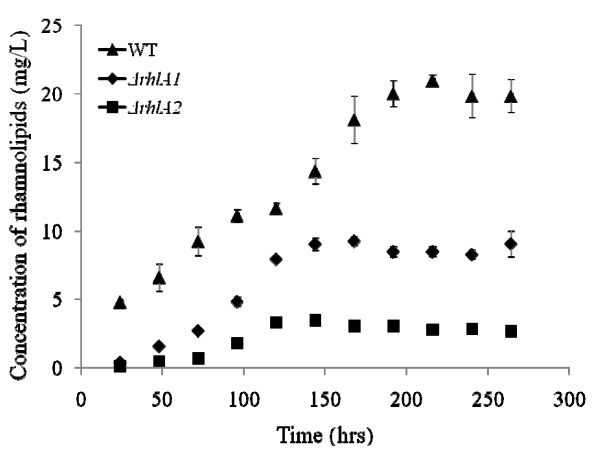
**Rhamnolipid production by single Δ*rhlA *mutants**. Total rhamnolipid production by the *B. thailandensis *E264 wild type strain and both single Δ*rhlA *mutant cultures grown in NB with glycerol (2%), as quantified by LC/MS. Each data point shows the mean of triplicate measurements. Error bars represent the SD. The double Δ*rhlA1rhlA2 *mutant does not produce any rhamnolipids.

The contribution to rhamnolipid production of the two identical *rhl *gene clusters found on the *B. thailandensis *genome was tested by creating single Δ*rhlA *mutants for each allele, as well as a double Δ*rhlA *mutant. These three mutants were then investigated for their ability to produce rhamnolipids (Figure 5). Five sets of replicates confirmed that the *B. thailandensis *Δ*rhlA1 *mutant produces more rhamnolipids than the Δ*rhlA2 *mutant. The rhamnolipids produced by each of these mutants are composed of the same congeners in the same proportions as the wild type strain and only a quantitative difference is observed. Intriguingly, total production of both single mutants do not equal to the production of the wild type strain. Finally, the double Δ*rhlA *mutant does not produce any detectable rhamnolipids.

### Swarming motility requires both *rhlA *alleles

**Figure 6:**
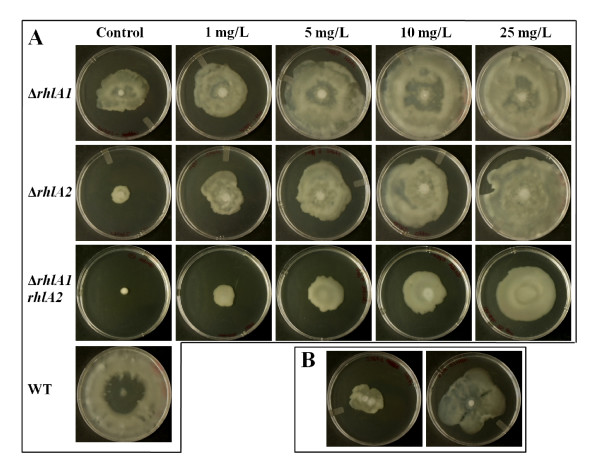
**Swarming phenotype restoration within the Δ*rhlA *mutants**. Swarm plates were incubated for 18 h at 30°C with *B. thailandensis *E264 wild type strain, both single Δ*rhlA *mutants as well as the double Δ*rhlA *mutant. Under these experimental conditions swarming motility is normally favored, as observed with the wild type strain. Experiments were done in triplicate. A) Swarming phenotype restoration of the Δ*rhlA *mutants with addition of 1, 5, 10 and 25 mg/L of exogenous purified rhamnolipids. B) Cross-feeding experimentation with both Δ*rhlA *single mutants. Left: mutants placed side-by-side; Right: mutants mixed before plating.

In *P. aeruginosa*, production of rhamnolipids is essential for expression of the multicellular behaviour called swarming motility [31]. It was therefore of interest to assess whether rhamnolipids are also important for this type of motility in *B. thailandensis*. Furthermore, since both *rhlA *alleles are functional and contributing to the production of rhamnolipids in this species, we wondered if the amount of biosurfactants produced by the single mutants would be sufficient to permit the swarming phenotype. Δ*rhlA1 *and Δ*rhlA2 *mutants of *B. thailandensis *were thus tested for their ability to swarm. Figure 6A (Control column) shows the swarming phenotype of the wild type strain as well as the single Δ*rhlA *mutants and the double Δ*rhlA *mutant. We observe that the single mutants have hindered swarming motility whereas the double mutant is incapable of such motility. Thus, one functional copy of *rhlA *does not provide enough rhamnolipid production to allow normal surface translocation on a semi-solid surface. Interestingly, the Δ*rhlA1 *mutant is capable of moving to a greater distance than the Δ*rhlA2 *mutant (Figure 6A). This observation concurs with the above results showing the superior rhamnolipid production by the Δ*rhlA1 *mutant compared to the Δ*rhlA2 *mutant (Figure 5). Finally, as expected, the double Δ*rhlA *mutant is incapable of any swarming.

To test whether swarming phenotype restoration is possible with our Δ*rhlA *mutants, swarm assays were performed with the addition of increasing concentrations of exogenous rhamnolipids. We observed that the Δ*rhlA1 *mutant requires less exogenous rhamnolipids to regain complete swarming motility compared to the Δ*rhlA2 *mutant, consistent with the finding that this latter mutant produces less rhamnolipids. These results indicate that a critical concentration of biosurfactant is necessary to enable bacteria to swarm. Accordingly, the double mutant requires much more exogenous rhamnolipids to restore this phenotype. Cross-feeding experiments with both Δ*rhlA *mutants were also performed to verify whether swarming phenotype could be regained. Interestingly, when the two mutants are mixed before plating, swarming is restored (Figure 6B, right), contrary to when mutants are simply spotted side-by-side (Figure 6B, left).

## Discussion

### *B. thailandensis *and *B. pseudomallei *harbor *rhlA/rhlB/rhlC *homologs for the biosynthesis of rhamnolipids

Looking through their sequenced genomes, we found that both *B. thailandensis *and *B. pseudomallei *harbor on their second chromosome two paralogous *rhl *gene clusters carrying genes highly similar to the *P. aeruginosa *genes *rhlA*, *rhlB *and *rhlC*, which are involved in the biosynthesis of rhamnolipids. Interestingly, in the latter species these three genes are arranged in two physically distant operons, while in the two *Burkholderia *species, they are part of the same gene cluster. The results presented here demonstrate that the purpose of these genes in *B. thailandensis*, and more than likely in *B. pseudomallei*, is for the production of rhamnolipids.

Genes that share similarities with efflux pumps and transporters are also present within the *rhl *gene clusters. There is at least one instance of an efflux system implicated in the transport of a biosurfactant. In the Gram-positive species *Bacillus subtilis*, YerP, a homolog to the resistance-nodulation-cell division (RND) family efflux pumps, was found to be implicated in surfactin resistance [32]. We propose that the other genes present within the *rhl *gene clusters are involved in the transport of rhamnolipids outside the cell; we are currently investigating this hypothesis.

Under our experimental conditions, *B. thailandensis *is capable of producing rhamnolipids with 3-hydroxy fatty acid moieties that are comprised of chains varying from C_10_-C_12 _to C_16_-C_16_. Such long lengths have not been reported for rhamnolipids produced by bacteria other than those of the *Burkholderia *species, with the exception of one publication reporting trace amounts of Rha-Rha-C_10_-C_14:1 _produced by *P. aeruginosa *57RP and another describing the production of a C_14_-C_10 _form by *P. chlororaphis *B-30761 [13,33]. Interestingly, the rhamnolipids produced by *B. thailandensis *are predominantly composed of dirhamnolipids, whereas monorhamnolipids and HAAs are only found in much smaller concentrations. Although the latter two are produced in smaller quantities by the bacteria, they are nevertheless comprised mostly of the corresponding molecule in the C_14_-C_14 _chain lengths. The dirhamnolipid versus monorhamnolipid ratio found in this species is approximately 13, whereas we observe a factor of only 4 in *P. aeruginosa*. One possible explanation is that, unlike *P. aeruginosa *which harbors *rhlA *and *rhlB *in one operon and *rhlC *in another, *Burkholderia *species code for the three enzymes from the same gene cluster, predicted to be an operon. We hypothesize this favors the simultaneous production of all the enzymes of the biosynthetic pathway; hence, RhlC would be present simultaneously and in the same stoichiometric ratio as RhlB, therefore favoring the immediate addition of the second L-rhamnose unto the monorhamnolipids. Our result adds *B. thailandensis *to the few bacterial species able to produce rhamnolipids, and shows that rhamnolipids produced by *Burkholderias *are more likely to contain longer side chains than those by *Pseudomonas *species, which are predominantly of the C_10_-C_10 _chain length.

The above mentioned facts are also true for the rhamnolipids produced by *B. pseudomallei*. More specifically, fatty acyl chains with carbon lengths of 12, 14 and 16 were observed in *B. pseudomallei *rhamnolipids, although only dirhamnolipids were detected. While production levels achieve 30 mg/L for *B. pseudomallei*, *B. thailandensis *can reach 80 mg/L under the same conditions (data not shown). Results of the present study further demonstrate that rhamnolipid congeners other than the previously described Rha-Rha-C_14_-C_14 _are also produced by this pathogen.

Inactivation of each of the two *rhlA *alleles confirmed that both *rhl *gene clusters contribute to the synthesis of rhamnolipids. Rhamnolipid production is observed even when one of the two alleles is not functional, suggesting that one copy does not depend on the other. However, the production levels attained by each of the Δ*rhlA *mutants show that the gene cluster containing the *rhlA2 *allele contributes about two and half more rhamnolipids than the *rhlA1 *allele cluster (Figure 5). Since the promoter sequences of the two *rhl *gene clusters only share approximately 270 bp directly upstream of both of the *rhlA *ATGs and therefore seem to have diverged, these results suggest that each cluster possesses its unique, differently controlled promoter, which is apparently found upstream of this conserved region. The biphasic shape of the wild-type rhamnolipid production curve supports this conclusion. Furthermore, the addition of both levels of production by the two clusters does not reach the wild type production level. This could be explained by some sort of positive retroaction where rhamnolipids stimulate global production and that the gene clusters are in fact interconnected. Also, it must be considered that the different rhamnolipid production levels attained by the Δ*rhlA *single mutants could also be associated to polar effects on the downstream genes that could possibly interfere with rhamnolipid biosynthesis.

The presence of two paralogous gene clusters is interesting since gene duplication is normally not favored within genomes, as one copy is generally more susceptible to mutations and/or inactivation. However, a duplication event might be preserved if it is immediately beneficial to the organism because of protein dosage effects, e.g. in variable environments [34,35]. We therefore extrapolate that the *Burkholderia *species which harbor twin *rhl *gene clusters have conserved both copies because it must be advantageous for these bacteria to produce extra quantities of rhamnolipids. The requirement of both *rhl *gene clusters for normal swarming motility supports this model (see below). The presence of a transposase of the mutator family in close proximity of one of the gene clusters (BTH_II1082) can also be indicative that a past duplication of an original single copy occurred and positive selection throughout evolution of some bacterial lineages conserved the paralogs.

### Long chain rhamnolipids from *Burkholderia: *effects on the CMC

Considering the length of the carbon chains of the fatty acid moiety of rhamnolipids produced by *Burkholderia *species, it was compelling to determine their effect on lowering the surface tension of water. A total rhamnolipid extract from *B. thailandensis *lowers the surface tension to 42 mN/m, with a CMC value of 225 mg/L. These values are higher than those traditionally reported for rhamnolipids produced by *Pseudomonas *species (typically around 30 mN/m and CMC in the order of 20 to 200 mg/L) [36]; however, it is only recently that HAAs have been discovered, as well as their efficacious surface tension-lowering potential [16]. Thus, we assume that results pertaining to surface tension properties of rhamnolipids published prior to this report could have been biased by a contamination with easily co-purified HAAs. For the purpose of the present study, we compared our results with those we have published for purified rhamnolipids and HAAs produced by *P. aeruginosa *PG201 [16]. The purified rhamnolipids from this strain lower surface tension to 40 mN/m with a CMC value of approximately 600 mg/L, while the HAA mixtures displays values of 29 mN/m with a CMC of approximately 800 mg/L. Consequently, it is clear that the longer chain rhamnolipids produced by *B. thailandensis *start forming micelles at a much lower concentration than *P. aeruginosa *rhamnolipids, 225 mg/L versus 600 mg/L. These values can be compared as the rhamnolipid mixture from *B. thailandensis *used for our tests contained only traces of HAAs. The effect of alkyl ester chain length of sophorolipids, a class of biosurfactants produced by *Candida bombicola*, has been studied with regards to micellization. The study reported a direct effect of carbon chain length on decreasing the CMC. Additional CH_2 _groups render the molecule more hydrophobic and thus facilitate micelle formation [37]. This might explain the lower CMC value obtained with the longer chain rhamnolipids produced by *B. thailandensis *in comparison to those obtained by *P. aeruginosa*.

### Both *rhlA *alleles are necessary for normal swarming motility

Swarming motility always involves biosurfactants. For example, serrawettin W2, a wetting agent produced by *Serratia liquefaciens*, is required for swarming motility in a nonflagellated mutant [38,39]. In regards to *P. aeruginosa*, biosurfactants such as rhamnolipids and HAAs are essential for swarming motility [7,16,40]. Only a few studies have reported on swarming motility of *Burkholderia *species, which is at least in part attributed to the lack of knowledge available regarding wetting agents produced by members of this genus. The swarming motility of *B. cepacia *has been observed, and the authors hypothesized that biosurfactants are involved [41]. We have also recently reported conditions under which *B. thailandensis *can swarm [42].

The present study demonstrates that swarming motility of a *B. thailandensis *double Δ*rhlA *mutant is completely prevented. This is in agreement with previous studies showing that inactivation of *rhlA *inhibits swarming by *P. aeruginosa *[16,40]. Furthermore, a mutation in any of the two *rhlA *genes hinders swarming of *B. thailandensis*, suggesting that a critical concentration of rhamnolipids is required and that the levels reached when only one of the two gene clusters is functional are not sufficient to allow the bacteria to completely overcome surface tension. The complementation experiment with exogenous addition of increasing concentrations of rhamnolipids further corroborates that there is indeed a critical concentration of biosurfactant necessary for *B. thailandensis *to swarm, and that both *rhl *gene clusters contribute differently to the total concentration of rhamnolipids produced. The cross-feeding experiment suggests that rhamnolipids produced by *B. thailandensis *diffuse to only a short distance in the agar medium surrounding the colony.

## Conclusions

The discovery that *B. thailandensis *is capable of producing considerable amounts of long chain dirhamnolipids makes it an interesting candidate for the production of biodegradable biosurfactants with good tensioactive properties. Furthermore, that this bacterium is non-infectious makes it an ideal alternative to the use of the opportunistic pathogen *P. aeruginosa *for the large scale production of these compounds for industrial applications. Finally, identification of the same paralogous *rhl *gene clusters responsible of the production of long chain rhamnolipids in the closely-related species *B. pseudomallei *might shed some light on the virulence mechanisms utilized by this pathogen during the development of infections.

## Methods

### Bacteria and culture conditions

The bacterial strains used in this study, *B. thailandensis *E264 (ATCC) [24] and *B. pseudomallei *1026b [43], were grown in Nutrient Broth (NB; EMD Chemicals) supplemented with 4% glycerol (Fisher) at 34°C on a rotary shaker, unless otherwise stated. *Escherichia coli *SM10 λpir (*thi-1 thr leu tonA lacY supE recA*::RP4-2-Tc::Mu Km^r ^λ*pir*) served as a donor for conjugation experiments and was grown in Tryptic Soy Broth (TSB) (Difco) under the same conditions [44]. When necessary, 150 μg/ml tetracycline or 100 μg/ml trimethoprim was added for *B. thailandensis *mutant selection.

To follow the production of rhamnolipids by *B. thailandensis *and its Δ*rhlA *mutants, cultures were grown in 50 ml of NB supplemented with 2% glycerol in 500 ml Erlenmeyer flasks at 37°C with gyratory shaking (240 rpm). For *B. pseudomallei*, cultures were carried out in 25 ml of NB supplemented with 4% glycerol in 250 ml Erlenmeyer flasks at 34°C with gyratory shaking (200 rpm).

### Rhamnolipid production and extraction

Cultures for high yield rhamnolipid production were grown in 200 ml of NB supplemented with 4% of glycerol or canola oil in 2 L Erlenmeyer flasks at 34°C with gyratory shaking (240 rpm). Extraction of total rhamnolipids was performed as described previously [16], with slight modifications. Briefly, cells were removed from the medium by centrifugation (13,000 × *g*, 15 min) and the supernatant acidified to pH 3-4 with concentrated HCl. The rhamnolipids were then extracted three times with 1/3 of the volume of ethyl acetate. The organic extract was then dried with anhydrous sodium sulfate and evaporated using a rotary evaporator. The oily residue was finally dissolved in methanol.

### Construction of ΔrhlA mutants

For the construction of single Δ*rhlA *mutants in *B. thailandensis*, a 464 bp fragment was amplified using primers rhlASVF and rhlASVR, containing *Xba*I and *Kpn*I restriction sites, respectively (Table 3). The PCR product was cloned by the means of its *Xba*I and *Kpn*I sites into the suicide vector pKNOCK-Tc [45]. The construct was transformed into competent *E. coli *SM10 cells by the heat shock method. The plasmid was then mobilized into *B. thailandensis *by mating and transformants were selected on TSB agar plates containing 50 μg/ml gentamicin, 15 μg/ml polymyxin B and 150 μg/ml tetracycline. To verify in which of the two *rhlA *alleles the homologous recombination took place, diagnostic PCRs were conducted using promoter-specific forward primers, rhlA1PF and rhlA2PF, as well as a common reverse primer, rhlAR, located at the end of the 3' regions of both *rhlAs*. Rhamnolipid production of mutants was also quantified (see below) and compared to typical wild type production values.

**Table 3:** Primers used in this study

Primer Name	Primer Sequence (5' to 3')
rhlASVF	GCTCTAGAAGACGGTCATCCTCGTGAAC^1^
rhlASVR	GGGGTACCCGGCAGCTTCGTCAGATAC^1^
rhlA1PF	GGAAATGGTCGATGGGTATG^2^
rhlA2PF	GGCGACGGATAGCGATAAG^2^
rhlAR	TCGTGTACTCGTCCAGCTC
rhlATp1F	GGCGGAATTCCGGCAGGTACTGCTCCGGCCGCATCGACAGGATCTGGTCCGAGCTCGAATTAGCTTCAAA
rhlATp1R	TGCCGCGGATCATGAAGCTGTACAACTACCGGTATCTGACGAAGCTGCCGGAGCTCGAATTGGGGATCTT
rhlA5'2F	GTGGTCGTGAAAGCGGAAT
rhlA5'2R	CGGCAGCTTCGTCAGATAC
rhlA3'3F	GACCAGATCCTGTCGATGC
rhlA3'3R	CTCGATCAGCGTCATCAGC

^1 ^Restriction sites designed into the primers are underlined.

^2 ^Primers are constructed upward of the consensus sequence of the two promoters.

To inactivate the second *rhlA *allele, targeted mutagenesis through natural transformation of PCR fragments was exploited [46]. Briefly, three fragments corresponding to the regions flanking the specific *rhlA *gene to be deleted and a trimethoprim resistance gene were joined by PCR. The 5' and 3' flanking regions of *rhlA *were amplified using primers rhlA5'2F and rhlA5'2R as well as rhlA3'3F and rhlA3'3R, respectively. The trimethoprim resistance marker was amplified from the pFTP1 plasmid (a gift from H. P. Schweizer, Colorado State University) using primers rhlATp1F and rhlATp1R [47,48]. Cells of the single Δ*rhlA *mutant were rendered competent using DM medium and then exposed to various concentrations of the mutagenic PCR fragment. Double Δ*rhlA *mutants were selected on TSB agar containing 150 μg/ml tetracycline and 100 μg/ml trimethoprim. The *B. thailandensis *Δ*rhlA *double mutant was confirmed by diagnostic PCR to verify proper recombination and insertion of the resistance marker. Absence of rhamnolipid production by LC/MS analysis also served as a confirmation.

### Preparation of culture samples for LC/MS analysis

To prepare samples for LC/MS analysis, the culture samples were firstly centrifuged to remove cells (16,000 × *g*, 15 min). To the cell-free supernatant was then added either 16-hydroxyhexadecanoic acid or deuterium-labeled 4-hydroxy-2-heptylquinoline (HHQ-D4) [49] as internal standards used for quantitative measurements, both at a final concentration of 10 mg/L. For the highly pathogenic *B. pseudomallei*, cell-free supernatants were obtained by centrifugation (16,000 × *g*, 15 min) followed by filtration on a 0.22 μm filter. Twenty μl of samples were injected for LC/MS analysis. Quantification was performed by integration of the pseudomolecular and the proper fragment ions and the use of dose-response calibration curves using purified rhamnolipids.

### Rhamnolipid analysis (LC/MS)

All rhamnolipid quantifications and analyses were performed using a Quattro II (Waters, Mississauga, Ontario, Canada) triple-quadrupole mass spectrometer in negative electrospray ionization mode coupled to an HP 1100 (Agilent Technologies, Saint Laurent, Quebec, Canada) high-performance liquid chromatograph (HPLC) equipped with a 4.6 × 50 mm 300SB-C3 Zorbax 5 μm (Agilent) reverse-phase column. The HPLC flow rate was set at 400 μl/min and was split to 10% by the means of a Valco Tee prior to being introduced into the mass spectrometer. An acetonitrile-water gradient containing 2 mM of ammonium acetate was used starting with 25% acetonitrile during the first 5 min, raised to 50% by 18 min and 100% by 19 min. This concentration was held until 22 min, where the initial concentration was resumed and kept until 26 min. Voltage of the capillary was set to 3.5 kV and cone voltage to 30 V. The temperature of the source block was kept at 120°C. Scan mass range was set from 130 to 940 Da. A calibration curve was performed to determine the long chain rhamnolipid response factor. During LC/MS/MS experimentation, fragmentation of the molecules were induced with argon serving as the collision gas at 2 × 10^-3 ^mTorr.

### Enzymatic hydrolysis of rhamnolipids - Naringinase

To study the rhamnolipid congeners produced by *B. thailandensis*, a modified protocol using enzymatic hydrolysis was employed, as described previously [30]. Briefly, 100 mg of extracted and purified rhamnolipids were suspended in 5 ml of 50 mM sodium acetate buffer, pH 4.1. To this solution was added 100 mg of naringinase from *Penicillum decumbens *(Sigma). The mixture was then kept at 50°C for 2 h with gyratory shaking (240 rpm), at which point 20 ml of buffer were added. After 24 h, another 150 mg of naringinase were added as well as 25 ml of buffer. The reaction was kept under these conditions for 8 days. A final 50 mg of naringinase in 20 ml of buffer were added to the mixture and was left for another 24 h. Thereafter, the solution was acidified to pH 3-4 using concentrated HCl and extracted three times with ethyl acetate. The fatty acid moieties generated by naringinase cleavage were then analyzed by LC/MS after the extract had been dried and evaporated.

### CMC - Surface tension assay

Critical micelle concentration and surface tension were measured by the du Noüy ring method [50] using a surface tensiometer (Fisher). The instrument was calibrated against water and assays were performed in triplicate at room temperature.

### Swarming motility

For swarming assays, cultures were grown overnight, diluted in fresh medium and subcultured until OD_600_~6.0 was reached. Swarm plates were prepared as follows: freshly autoclaved medium consisting of NB supplemented with 0.5% dextrose (Fisher) and 0.5% Bacto-agar (Difco) was poured into standard Petri dishes and dried under laminar flow for 30 min, as before [42]. Immediately following the drying period, plates were inoculated at their center with 5 μl of bacterial culture and placed at 30°C.

For swarming phenotype restoration, 1, 5, 10 and 25 mg/L of purified *B. thailandensis *E264 rhamnolipids were deposited (10 μl) at the center of respective plates and left to dry for 15 minutes before spot inoculation with swarming-deficient Δ*rhlA *mutant strains. For cross-feeding experiments, either equal parts of the cultures were mixed before being plated at the center on the swarm plate, or cultures were simply spotted side-by-side.

## Authors' contributions

ED and DD designed the experiments. DD carried out all experimental procedures and analyzed the data. FL provided critical knowledge in LC/MS experimentation. DEW provided *B. pseudomallei *samples for LC/MS analysis. DD wrote the manuscript. FL and ED corrected the manuscript. All authors read and approved the final manuscript.
